# Chitosan/siCkip-1 biofunctionalized titanium implant for improved osseointegration in the osteoporotic condition

**DOI:** 10.1038/srep10860

**Published:** 2015-06-04

**Authors:** Li Zhang, Kaimin Wu, Wen Song, Haiyan Xu, Ran An, Lingzhou Zhao, Bin Liu, Yumei Zhang

**Affiliations:** 1 State Key Laboratory of Military Stomatology, Department of Prosthetic Dentistry, School of Stomatology, The Fourth Military Medical University, No. 145 West Changle Road, Xi’an 710032, China; 2 State Key Laboratory of Military Stomatology, Department of Periodontology, School of Stomatology, The Fourth Military Medical University, No. 145 West Changle Road, Xi’an 710032, China; 3 Department of Stomatology, 401 Military Hospital, Qingdao 266071, China; 4 State Key Laboratory of Military Stomatology, Laboratory Animal Center, School of Stomatology, the Fourth Military Medical University, No. 145 West Changle Road, Xi’an 710032, China

## Abstract

Biofunctionalization with siRNA targeting the key negative modulators of bone turnover involved in the molecular mechanism of osteoporosis, such as casein kinase-2 interacting protein-1 (Ckip-1), may lead to enhanced Ti osseointegration in the osteoporotic condition. In this study, even siRNA loading was accomplished by the thermal alkali (TA) treatment to make the Ti ultrahydrophilic and negatively charged to facilitate the physical adsorption of the positively charged CS/siR complex, designated as TA-CS/siR. The intracellular uptake of the CS/siR complex and the gene knockdown efficiency were assessed with bone marrow mesenchymal stem cells (MSCs) as well as the green fluorescent protein (GFP) expressing H1299 cells. *In vitro* osteogenic activity of TA-CS/siCkip-1 targeting Ckip-1 was assessed with MSCs. *In vivo* osseointegration of TA-CS/siCkip-1 was assessed in the osteoporotic rat model. TA-CS/siR showed excellent siRNA delivery efficiency and gene silencing effect. TA-CS/siCkip-1 significantly improved the *in vitro* osteogenic differentiation of MSCs in terms of the enhanced alkaline phosphatase and collagen product and extracellular matrix mineralization, and led to dramatically enhanced *in vivo* osseointegration in the osteoporostic rat model, showing promising clinical potential for the osteoporotic condition application. TA-CS/siR may constitute a general approach for developing the advanced Ti implants targeting specific molecular mechanism.

Titanium (Ti) implants have been widely applied as bone substitutes, orthopedic and dental implants, as well as many other biomedical appliances due to their good mechanical property, excellent biocompatibility and unique osseointegration capacity^1^. A high 10-year survival rate of about 95% has been achieved for the Ti based bone implants in healthy patients^2^. However, they still suffer from deficient bioactivity that may lead to implant failure, especially when encountering some complicated conditions that do not favor the osseointegration establishment. For example, osteoporosis, one of the major public health problems around the world characterized by excessive bone loss and low bone formation^3^, can severely compromise the primary stability and osseointegration establishment of Ti implant^4^. Then specifically designed Ti implant with sufficient osseointegration establishing ability in the osteoporotic condition is urgently in need. To that end, biofunctionalization represents a promising approach^5^,^6^.

Currently, biofunctionalization of biomaterials has been mainly conducted with extracellular matrix components, growth factors, peptides, etc., which do not directly touch the molecular mechanism underlying the osteoporosis occurrence and development thereby generating limited biological effect^6^. Biofunctionalization to target the key molecular events in osteoporosis shall more robustly improve the osseointegration in the osteoporotic condition. It is found that some negative bone turnover modulators play key roles in the osteoporosis occurrence and development thanks to the development in the molecular mechanism study of osteoporosis. Meanwhile, short interfering RNA (siRNA, siR) delivery, a highly efficient and specific gene silencing technology, has been greatly advanced, which is considered to be a promising approach for the treatment of various diseases^7^. Then it is feasible to develop the siRNA biofunctionalized Ti implant targeting the negative bone turnover modulator involved in osteoporosis for enhanced osseointegration in the osteoporotic condition. For example, casein kinase-2 interacting protein-1 (Ckip-1) is found to be involved in osteoporosis by negatively regulating the bone turnover via specifically upgrading the E3 ligase activity of Smurf1^8^. The Ckip-1 knockdown by siRNA notably increases the bone mass and enhances structure of trabecular bone in both healthy and osteoporotic rats^9^,^10^. Thus, Ckip-1 siRNA (siCkip-1) is a good candidate for the implant biofunctionalization.

Though there are spgeveral reports on the siRNA biofunctionalization of tissue engineering scaffolds^11^,^12^,^13^,^14^ and our group have reported the microRNA functionalized Ti implant^15^,^16^, the siRNA functionalized Ti implant has not been reported yet. There are three crucial issues to be considered when developing the siRNA biofunctionalized Ti implant, namely the suitable siRNA vector selection, loading capacity increase of the Ti implant as well as siRNA loading strategy. A vector of high delivery efficiency and simultaneously satisfactory biocompatibility, safety and storability is required. The commercial cationic lipid vector with high delivery efficiency is not an ideal choice for biofunctionalization application due to cytotoxicity^17^. Chitosan, as a natural degradable cationic polymer with nice merits of biocompatibility and cost economy, has recently been extensively studied as oligonucleotide vector. The results show that chitosan can flexibly bring abundant amount of siRNA with ignorable cytotoxicity^18^,^19^, thus constituting a good vector candidate. It would be ideal to increase the Ti implant loading capacity and facilitate an even and robust siRNA loading via one simple step. Excitingly, the thermal alkali (TA) treatment may cater for these requirements. The uniform microporous/nanofibrous structure on Ti generated by the TA treatment^20^,^21^ shall lead to increased loading capacity, and meanwhile its ultrahydrophilic and negatively charged nature^20^,^21^ will facilitate the adsorption and retention of the positively charged chitosan/siRNA (CS/siR) complex^22^. It is noteworthy that the TA treated Ti *per se* shows bone formation favoring property to stimulate hydroxyapatite deposition and promote osteoblast adhesion and proliferation^20^,^23^. Thus, excellent bioactivity and satisfactory osseointegration can be expected from the CS/siCkip-1 biofunctionalized TA Ti implant (TA-CS/siCkip-1). In this study, TA-CS/siCkip-1 was developed, whose *in vitro* effect on the primary bone marrow mesenchymal stem cells (MSCs) and *in vivo* osseointegration in the osteoporostic rat model were evaluated.

## Materials and Methods

Commercial pure Ti foils (10 × 10 × 1 mm^3^) and Ti rods (ø1.56 mm × 10 mm) were provided by Northwest Institute for Nonferrous Metal Research, China. Chitosan (150 kDa, 95% deacetylation) was obtained from HEPPE MEDICAL, Germany. siCkip-1 (sense: 5′-GGACUUGGUAGCAAGGAAATT-3′, antisense: 5′-UUUCCUUGCUACCAAGUCCTT-3′) labeled with Cy3 or not, negative control siRNA duplex targeting murine TNF-α (siNC, sense: 5’-pGUCUCAGCCUCUUCUCAUUCCUGct-3’, antisense: 5’-AGCAGGAAUGAGAAGAGGCUGAGACAU-3’), siRNA duplex targeting enhanced green fluorescent protein (EGFP) (siGFP, sense: 5’-GACGUAAACGGCCACAAGUUC-3’, antisense: 5’-ACUUGUGGCCGUUUACGUCGC-3’) labeled with Cy3 or not, and the real-time polymerase chain reaction (real-time PCR) primer were bought from Dharmacon. Hoechst Dye, Bovine serum albumin (BSA) and sodium dodecyl sulfate (SDS) were bought from Invitrogen Corporation. Sirius red, saturated picric acid and alizarin red, paraformaldehyde, β-glycerophosphate, ascorbic acid, dexamethasone and pelltobarbitalum natricum were obtained from Sigma. BCA protein quantification kit was obtained from Novagen. PrimeScript RT reagent kit and SYBR Premix Ex Taq™ II were obtained from TaKaRa. RPMI media, fetal bovine serum (FBS), penicillin/streptomycin, Geneticin, α minimum essential medium (α-MEM), fetal calf serum (FCS) and phosphate buffered saline (PBS) were purchased from Gibco. Cell count kit-8 (CCK-8) and BCIP/NBT ALP color development kit were bought from Beyotime.

The Ti samples were wet polished with the SiC paper from mesh 400 to mesh 1200 (designated as PT), followed by ultrasonic cleaning in acetone, alcohol and deionized water successively and then dried. For TA treatment, the PT samples were soaked in 5 M sodium hydroxide aqueous solution at 60 °C for 24 hours. Afterwards, they were ultrasonically washed with deionized water thoroughly and finally dried at 60 °C in air, with the samples formed designated as TA.

The CS/siR complex was formulated according to a previous report^24^. In concise, CS was totally dissolved in 0.2 M sodium acetate buffer (0.8 mg/ml, pH 5.5) and mixed vigorously with 20 μM siRNA at an N/P ratio of ~60. The hydrodynamic size and the zeta potential of the CS/siR complex were examined by dynamic light scattering (DLS) using a Malvern zeta sizer (Malvern Instrumentation Co.) according to the manufacturer’s instruction. The particle size and zeta potential values were calculated from five measurements per sample. To load the CS/siR complex onto the TA Ti samples, the TA Ti samples were immersed in 250 μl of the CS/siR complex solution at 4 °C for 24 hours. Afterwards, the Ti samples were rinsed gently with acetate buffer and dried at 4 °C to finally get the CS/siR biofunctionalized TA Ti implant (TA-CS/siR).

The surface morphology of the Ti samples was observed by scanning electron microscopy (SEM, Hitachi S-4800). The surface of Ti-CS/siCkip-1 with Cy3 labeled siCkip-1 was scanned by confocal laser scanning microscopy (CLSM, Olympus FV1000). The surface wettability of the Ti samples (5 samples per group) was evaluated. The images of the pure water droplet on the Ti samples were captured 10 seconds after contacting by an imaging analysis microscope (Camscope, Sometech Inc.) and the contact angle was measured by analyzing the drop shape using the DSA1 software (KRUSS).

To assess the protein absorption ability of the Ti samples, they (5 samples per group) were immersed in 1 ml of 0.5 mg/ml BSA solution for 24 hours. Then the adsorbed proteins were eluted by 250 μl of 1% SDS and the BSA amount in the eluted solution was quantified by the BCA protein quantification kit.

The CS/siGFP complex intracellular uptake and gene knockdown efficiency were assessed with siGFP and the GFP expressing H1299 cells that were maintained in RPMI media containing 10% FBS, 1% penicillin/streptomycin and 0.2% Geneticin. The CS/siCkip-1 complex intracellular uptake and gene knockdown efficiency were assessed with siCkip-1 and MSCs.

The osteogenic ability of TA-CS/siCkip-1 was evaluated with the MSCs. The MSCs were isolated as previously reported^15^,^25^ and maintained in α-MEM with 10% FCS in a humidified atmosphere with 5% CO_2_ at 37 °C. The animal experiment was approved by the Animal Research Committee of the university (No.2013-kq-004) and conducted in accordance with the international standards on animal welfare. The medium was changed every 3 days. The cells at passage 2–4 were used in the experiments.

The H1299 cells were seeded on TA-CS/siGFP with Cy3 labeled siGFP placed in the 24-well cell culture plate at a density of 2 × 104 cells/well and cultured for 24 hours. The MSC cells were seeded on TA-CS/siCkip-1 with Cy3 labeled siCkip-1 placed in the 24-well cell culture plate at a density of 2 × 104 cells/well and cultured for 24 hours. Afterwards, the cell cultures were fixed with 1% paraformaldehyde and washed in PBS. The cell nucleus was stained with the Hoechst Dye. Finally, the fluorescence signals of Hoechst and Cy3 were observed by confocal laser scanning microscopy.

With TA-CS/siGFP and the H1299 cells, the knockdown efficiency of TA-CS/siR can be easily assessed by monitor the GFP expression of H1299 cells. The H1299 cells were seeded on the Ti samples placed in the 24-well cell culture plates at a density of 2 × 10^4^ cells/well. After 3 days of culture, the GFP expression of the H1299 cells was captured by inverted fluorescence microscopy (DMI3000B, Leica). The GFP fluorescent intensity was analyzed by Image J.

The gene knockdown efficiency of siCkip-1 on the MSCs was evaluated using the real-time PCR. Briefly, 48 hours after the cell seeding, the total RNA was isolated using Trizol reagent (Invitrogen). Then, RNA of 2 μg from each sample was converted to cDNA using a PrimeScript RT reagent kit (TaKaRa). The analysis was performed on the CFX96™ Real Time RT-PCR System with SYBR Premix Ex Taq™ II (TaKaRa). The Ckip-1 expression was normalized to that of the housekeeping gene glyceraldehyde-3-phosphate dehydrogenase (GAPDH). The primers pairs were as follows: Ckip-1 (sense: 5’-CTATCCCAGAGGGACACGC-3’, antisense: 5’-ATCTCCCAGTCCCTGAACCT-3’, GAPDH: (sense: 5’-GGCACAGTCAAGGCTGAGAATG-3’, antisense: 5’-ATGGTGGTGAAGACGCCAGTA-3’).

The MSCs were seeded on the Ti samples (5 samples per group at each time point) at a density of 2 × 10^4^ cells/well. At 1, 4 and 7 days, the cell proliferation was evaluated quantitatively using CCK-8. Briefly, the culture medium was removed and the cells were rinsed slightly with PBS. Then, 360 μl medium and 40 μl CCK-8 solution was added to each well and incubated for 2 hours at 37 °C. Finally, the supernatant was collected to determine the absorbance at 450 nm using a spectrophotometer (Bio-Tek).

The MSCs were seeded on the Ti samples placed in 24-well plates at a density of 2 × 10^4^/well. For osteogenic induction, 3 days post-seeding the cell culture medium was shifted to the osteogenic medium containing 10 mM β-glycerophosphate, 50 μg/ml ascorbic acid and 10 nM dexamethasone. At predetermined time points, the alkaline phosphatase (ALP) production, collagen secretion and extracellular matrix (ECM) mineralization were measured to evaluate the osteogenic differentiation of MSCs on the Ti samples (5 samples per group for each assay). After 7 days of culture in the osteogenic medium, the Ti samples with cells were washed with PBS and fixed with 4% paraformaldehyde. The ALP production was stained by the BCIP/NBT ALP color development kit for 15 minutes and the images were taken. The collagen secretion on the Ti samples was quantified using a method described before^26^. After 14 days of culture in the osteogenic medium, the Ti samples with the cells were washed, fixed and stained with 0.1wt% sirius red in saturated picric acid for 18 hours. The unbound stain was washed with 0.1 M acetic acid before the images were taken. For quantitative analysis of the collagen production, 0.2 M NaOH/methanol (1:1) was used to dissolve the stain to measure the absorbance at 540 nm. ECM mineralization was evaluated by alizarin red staining^27^. Briefly, after incubation for 21 days in the osteogenic medium, the Ti samples were washed with PBS, fixed and stained with 1 wt% alizarin red for 10 minutes. After thorough washing with distilled water, images were taken. To quantify the ECM mineralized nodules, 10% cetylpyridinium chloride in 10 mM sodium phosphate was used to dissolve the stain and the absorbance values at 620 nm were determined.

The animal experiment was approved by the Animal Research Committee of the Fourth Military Medical University (No.2013-kq-004) and conducted in accordance with the international standards on animal welfare. Female Sprague-dawley rats weighing 200~230 g were used in this study. They were kept in individual cages and offered with standard diets. After 10 days of adaptation to the environment, they underwent bilateral ovariectomy (OVX) and three months later the osteoporotic model was established. The SHAM control only had the same mass of fat tissue excised during the surgery. To confirm the successful establishment of osteoporosis model, three months after the OVX and SHAM surgeries, 10 rats of the OVX and SHAM group were sacrificed to obtain the femurs for micro-CT scanning (Inveon CT, Siemens). The quantitative parameters including bone volume ratio (BV/TV), trabecular thickness (Tb.Th), trabecular separation (Tb.Sp) and trabecular number (Tb.N) were obtained. Once established, the osteoporotic rats were randomly divided into four groups to receive TA-CS/siCkip-1 and the controls including TA-CS/siNC, TA-CS and TA. The distal femur metaphysis was chosen as the implantation site. Every rat received two implants of the same group. Briefly, the rats were anesthetized by intraperitoneal injection of 1% pelltobarbitalum natricum (4 mg/kg) and fixed in the supine position. An incision of about 15 mm long was made in the medial knee and the muscle tissue was separated to expose the femur bone surface. A hole parallel to the long axis of the femur was made using a 1.56 mm rotary drill cooled with saline solution. Finally, the implants were pressed into the medullary cavity until they reach the site below the growth plate and then the muscle tissue and skin was sutured separately. Antibiotics were administrated for 3 consecutive days post-surgery.

Micro-CT analysis was also conducted to assess the new bone formation around the implants. At 3 months after implantation, the animals were sacrificed to retrieve the femurs with implants in (5 samples per group). Region of interest (ROI) was defined as a ring with a 200 μm radius starting from the implant surface in the coronal plane. The two- (2-D) and three-dimensional (3-D) views of the implant with the surrounding new bone were reconstructed. The quantitative parameters including BV/TV, Tb.Th, Tb.Sp and Tb.N of the ROI were obtained.

After being harvested 1 and 3 months after implantation, the femurs (5 per group) with implants in were fixed dehydrated and embedded in methylmethacrylate. Then blocks were cut parallel to the long axis of femurs with running water cooling and sections of about 70 μm in thickness were obtained. The sections were polished and stained with 1% acid fuchsine and 0.5% saturated picric acid (Van-Greson staining, VG staining) for histological observation by stereomicroscopy (M80, Leica).

After harvest 3 months after implantation, the femurs with implants in (5 per group) were fixed with 4% paraformaldehyde for one week. They were cleaned by running water, dehydrated in gradient ethanol (50–100%) and embedded in methylmethacrylate. Then a macro-cutting and grinding system (SP1600&SP2600, Leica) was used to cut the femurs into sections parallel to the long axis of the implants. Finally, the cross-section samples of implant/bone interface were treated with conductive coating and scanned by SEM. The line-profiles of Ti, C, O, Ca P in the direction perpendicular to the interface were measured by energy dispersive X-ray spectroscopy (EDX, Hitachi).

Biomechanical pull-out test was carried out to evaluate the bonding strength between implant and bone with a material testing system (Shimadzu, AGS-10kNG). Immediately after being harvested 1 and 3 months after implantation, the distal metaphysis of the femurs with implants in (5 per group) was shaved to expose 3 mm long of the implant. The exposed implant part was embedded with self-curing plastic for fixing it to the testing machine. During the test, a pulling force was given along the long axis of the femur at the speed of 1 mm/min and the load-displacement curve was recorded. The maximum pull-out force can be obtained from the curve and the shear strength can be calculated accordingly.

Data were expressed as mean ± standard deviation (SD). Statistical analyses were performed using the statistics package SPSS 17.0 (SPSS, USA). Comparison among groups was made using the one way ANOVA and Student-Newman-Keuls *post hoc* tests. Difference was considered to be significant at *p* < 0.05.

## Results

**Figure 1:**
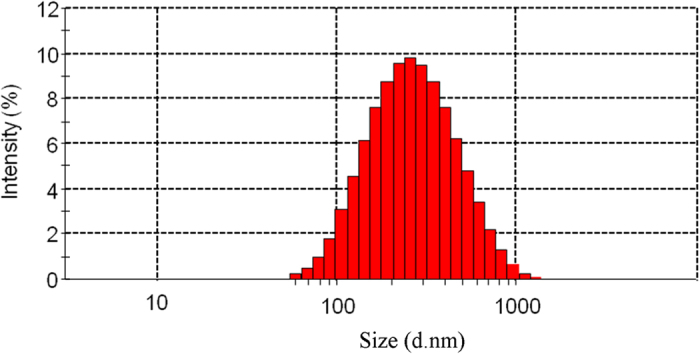
Particle size of CS/siR complex.

The hydrodynamic size of CS/siR complex was measured (Fig. 1). The size of the CS/siR complex varies from 100 to 1000 nm with an average value of ~235.9 ± 25.6 nm. The mean zeta potential of CS/siR complex was about 13.7 ± 2.77 mV.

**Figure 2:**
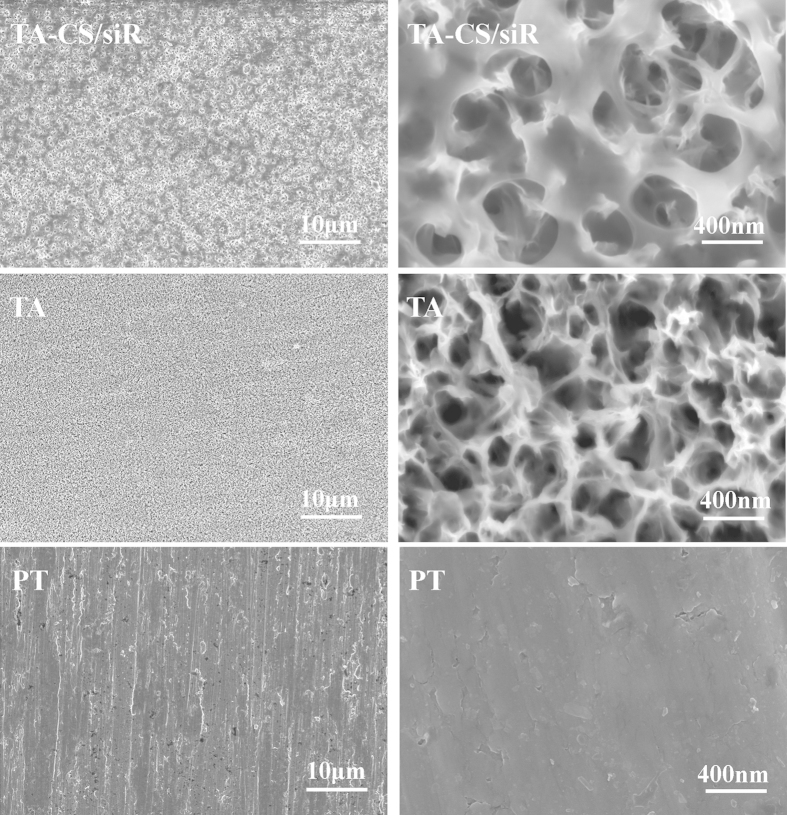
SEM observation of TA-CS/siR, TA and PT.

The surface morphology of PT, TA and TA-CS/siR was displayed by SEM (Fig. 2). PT owns a relative smooth surface structure with normal grinding scratch. TA possesses a microporous/nanofibrous network structure with greatly enlarged surface area compared to PT and the pore diameters range from 100 to 400 nm, which were in consistence with the findings in previous literatures^28^,^29^. TA-CS/siR formed by 24 hour soaking in the CS/siR complex solution is evenly covered with amorphous CS/siR complex. The CS/siR complex could enter into the microporous and the interfibrous space.

**Figure 3:**
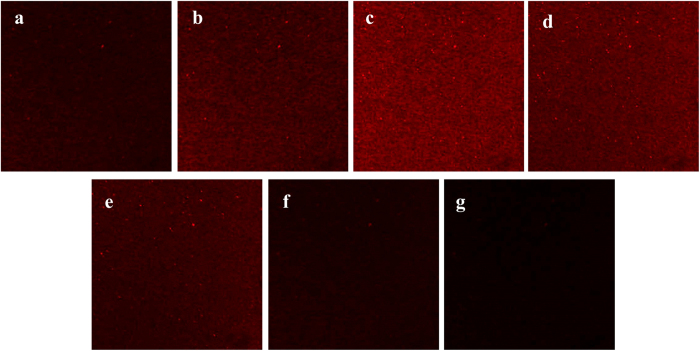
Fluorescence CLSM images of TA-CS/siCkip-1 with the Cy3-labeled siCkip-1 (red colour) from top to bottom (**a-g**) with an interlayer distance of 400 nm.

To observe the distribution of the CS/siR complex on TA-CS/siR, the Cy3-labeled siCkip-1 was used to fabricate the TA-CS/siR sample followed by inspection by CLSM (Fig. 3). The TA-CS/siR surface was scanned layer by layer from the surface to bottom of the coating with a 400 nm interlayer interval (Fig. 3a-g). The fluorescence images further corroborated that the CS/siR complex disturbed evenly on the Ti surface and went into deeper area of the microporous TA structure. The thickness of the CS/siR complex layer deposited on the TA surface is estimated to be about 2000 nm.

**Figure 4:**
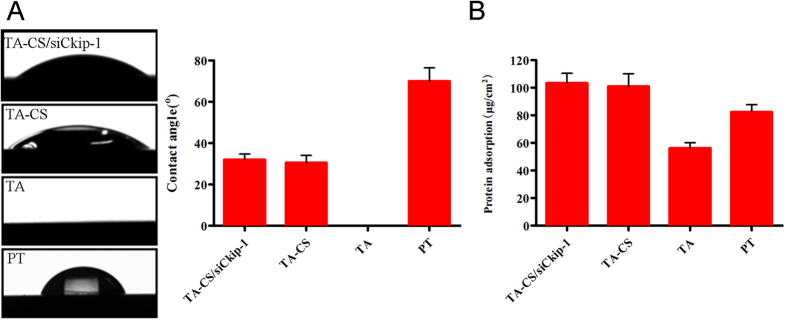
(**A**) Water contact angle measured 10 seconds after contacting the Ti surfaces and (**B**) BSA protein adsorption amount measured after 24-hour immersion in 1 ml BSA solution of 0.5 mg/ml for TA-CS/siCkip-1, TA-CS, TA and PT.

The water contact angle of TA-CS/siCkip-1, TA-CS, TA and PT was assessed (Fig. 4). The water contact angle for PT was 65 ± 8°. In respect of TA, the water droplets spread very quickly once contacting the Ti surface, resulting in undetectable contact angle. TA-CS/siCkip-1 and TA-CS generated water contact angles of 30 ± 3°, which was much higher compared to that of TA but still significantly lower than that of PT.

The protein adsorption ability of the samples was assessed by immersion in the BSA solution for 24 hours (Fig. 4). TA induced obviously lower protein adsorption amount (45 ± 5 μg/cm^2^) compared to PT (80 ± 8 μg/cm^2^). However, TA-CS and TA-CS/siCkip-1 resulted in significantly enhanced protein adsorption amounts (100 ± 12 μg/cm^2^) that were even higher than those induced by PT.

**Figure 5:**
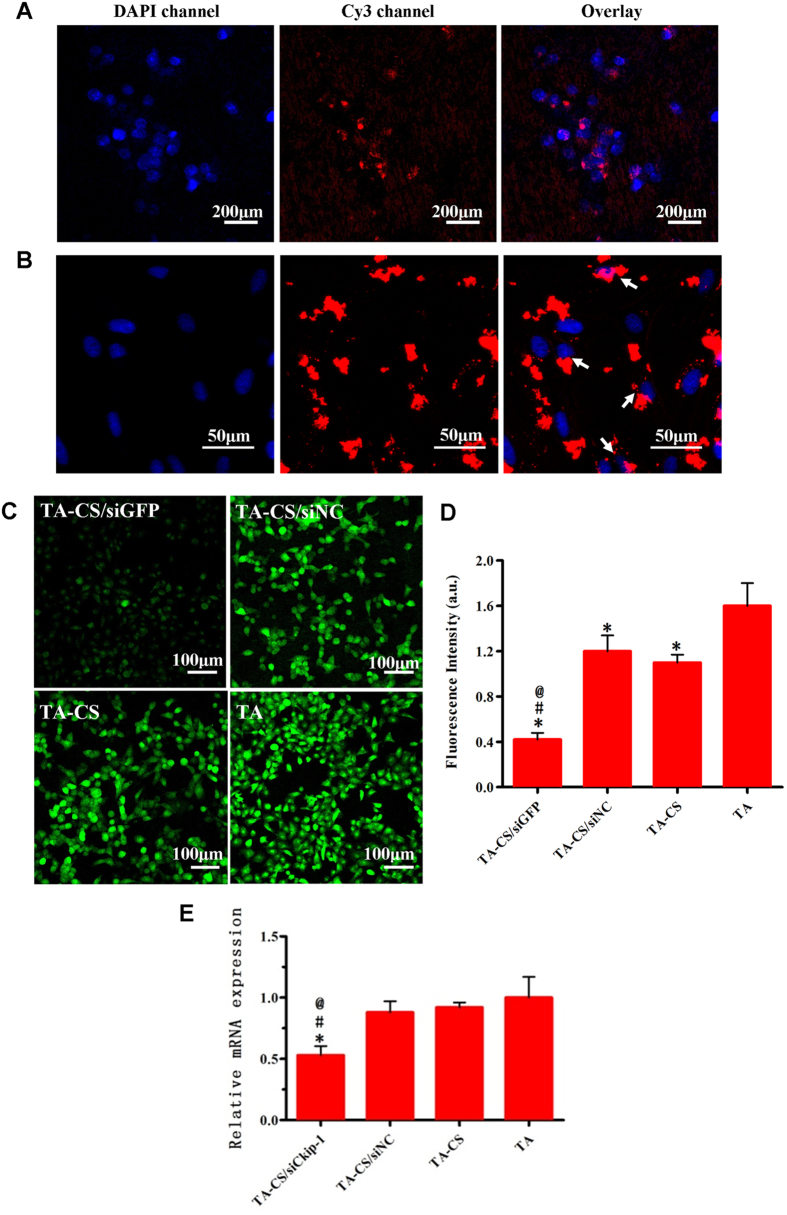
(**A**) Cellular internalization of siGFP (red) from the Ti surfaces. The cell nucleus was stained by Hoechst Dye (blue). (**B**) Cellular internalization of siCkip-1 (red) from the Ti surfaces. The cell nucleus was stained by Hoechst Dye (blue). (**C**) GFP expression by the H1299 cells after 3-day culture on the Ti samples. (**D**) The average GFP fluorescent intensity of cells in Fig. 5, C by Image J. *p < 0.05 *vs* TA, #*p* < 0.05 *vs* TA-CS, @*p* < 0.05 *vs* TA-CS/siNC. (**E**) Relative Ckip-1 mRNA expression levels measured by real-time PCR 48 hours after culture on the Ti samples. *p < 0.05 *vs* TA, #*p* < 0.05 *vs* TA-CS, @*p* < 0.05 *vs* TA-CS/siNC.

The H1299 cells were seeded on TA-CS/siGFP fabricated with the Cy3-labeled siGFP and 24 hours later the cellular internalization of CS/siGFP complex was observed by CLSM (Fig. 5). The fluorescent signals of siGFP (red) mainly locate surrounding the cell nucleus (blue). On the other area of Ti surface without cells, the fluorescent signals of siGFP are nearly undetectable, indicating a successful cellular internalization of the CS/siGFP complex. Similar phenomenon was observed for the intracellular uptake of asiCkip-1 by MSCs (Fig. 5). The findings were in consistence with results in a previous report^30^.

To assess the gene knockdown efficiency of TA-CS/siR, the GFP expressing H1299 cells were cultured on TA-CS/siGFP for 3 days to observe the GFP expression change (Fig. 5). The GFP expression by H1299 on TA-CS/siGFP was significantly lower compared to that on the three controls. The fluorescent intensity of the images in Fig. 5 was semi-quantitatively analyzed by ImageJ (Fig. 5), which more clearly displays that TA-CS/siGFP induced significantly lower GFP expression compared with other three controls, about one tenth of the TA control. Meanwhile, an obvious GFP expression knockdown was also observed on TA-CS and TA-CS/siNC compared to PT, which shall be related to the non-specific gene knockdown effect of CS. To assess the gene knockdown efficiency of TA-CS/siCkip-1 on MSCs, the relative expression of Ckip-1 by MSCs on different substrates 48 hours after incubation was evaluated using the real-time PCR (Fig. 5). The intracellular Ckip-1 mRNA level in MSCs was significantly down-regulated by TA-CS/siCkip-1 compared to the other control groups, being about 40% lower than that in the siNC control group.

**Figure 6:**
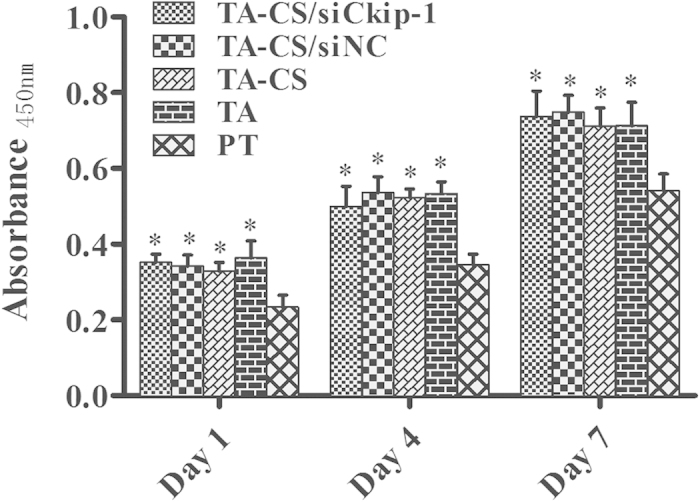
Proliferation of MSCs on the Ti samples measured by the CCK-8 assay at 1, 4 and 7 days. ^*^p < 0.05 compared with PT.

The proliferation of MSCs on the Ti samples measured by the CCK-8 assay is shown in Fig. 6. The cell proliferation on all Ti samples increased with the incubation time from 1 to 7 days. Compared to PT, TA led to significantly higher cell proliferation. No apparent difference in cell proliferation was observed among TA, TA-CS, TA-CS/siNC and TA-CS/siCkip-1 at all time slots, demonstrating that the biofunctionalization process did not compromise the cytocompatibility of the Ti implant.

**Figure 7:**
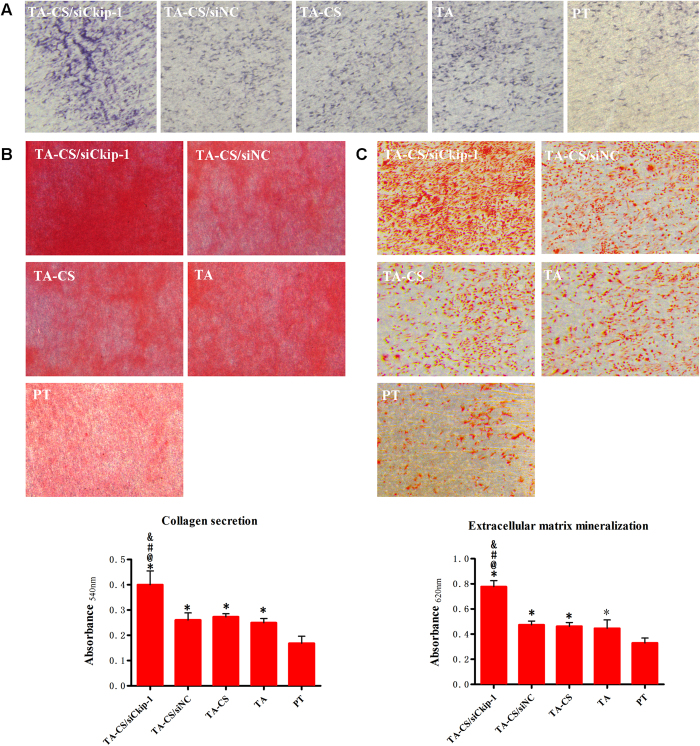
(**A**) ALP staining after 7 days of osteogenic induction; (**B**) Collagen staining and semi-quantitative results after 14 days of osteogenic induction and (**C**) ECM mineralization staining and semi-quantitative results after 21 days of osteogenic induction on The Ti samples. *p < 0.05 *vs* PT, @*p* < 0.05 *vs* TA, #*p* < 0.05 *vs* TA-CS, &*p* < 0.05 *vs* TA-CS/siNC.

The osteogenic differentiation of MSCs on the Ti samples was evaluated in terms of the ALP and collagen secretion and ECM mineralization, which showed similar trend among the different Ti samples (Fig. 7). TA-CS/siCkip-1 generated much more ALP and collagen product and better ECM mineralization than all the controls. TA, TA-CS, TA-CS/siNC induced similar ALP and collagen product and ECM mineralization which were higher than those induced by PT. According to the semi-quantitative data, the collagen amount and ECM mineralization yielded by TA-CS/siCkip-1 was about 1.5 times of those on TA, TA-CS and TA-CS/siNC and 2 times of those on PT.

Three months after the OVX surgery, the micro-CT analysis was performed to confirm the successful establishment of the osteoporotic model, which is shown in Figure S1. The sagittal 2-D graphs and the 3-D reconstructed views of the distal femur metaphysis show that the OVX rats have significantly lower bone mineral density, looser trabecular bone and larger gap between trabecular bones than the SHAM control. The quantitative data display that the OVX rats have significantly lower BV/TV and TB.N but higher TB.Sp compared to the SHAM control. The data explicitly demonstrate the successful establishment of the osteoporotic model.

**Figure 8:**
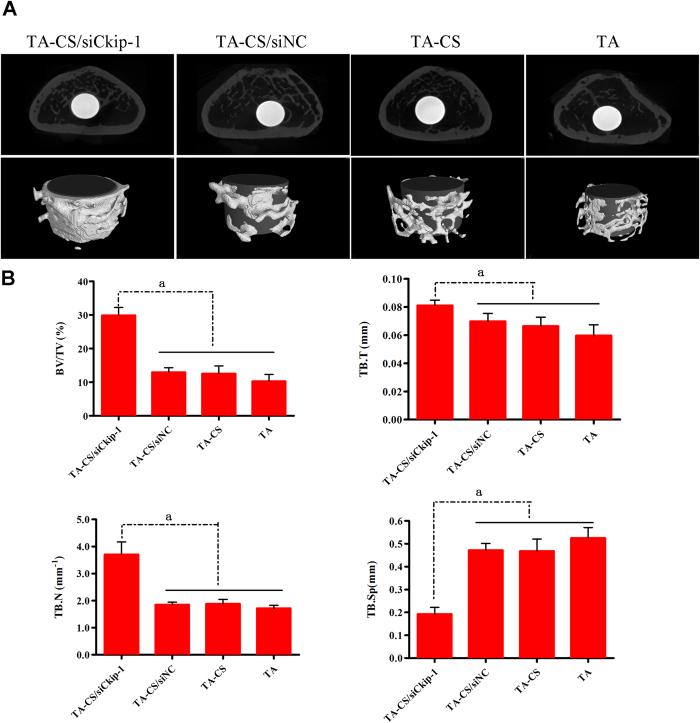
(**A**) Transverse 2-D images and 3-D reconstructed views of the Micro-CT analysis to show the formation of new bone around the Ti implants. (**B**) The quantitative data obtained from the micro-CT analysis including BV/TV, TB.Th, TB.N and TB.Sp. ^*^p < 0.05.

Three months after implant insertion in the OVX rats, the new bone formation around the Ti implants was assessed by the micro-CT scanning (Fig. 8). The transverse 2-D graphs and the 3-D views show the details of the bone response around the implant (Fig. 8). The new bone formation around TA-CS/siCkip-1 is far better than the controls. A continuous and thick layer of new bone is observed around TA-CS/siCkip-1. The quantitative analysis reveals that TA-CS/siCkip-1 can enormously increase BV/TV and the trabecular number, and decrease the trabecular spacing (Fig. 8B).

**Figure 9:**
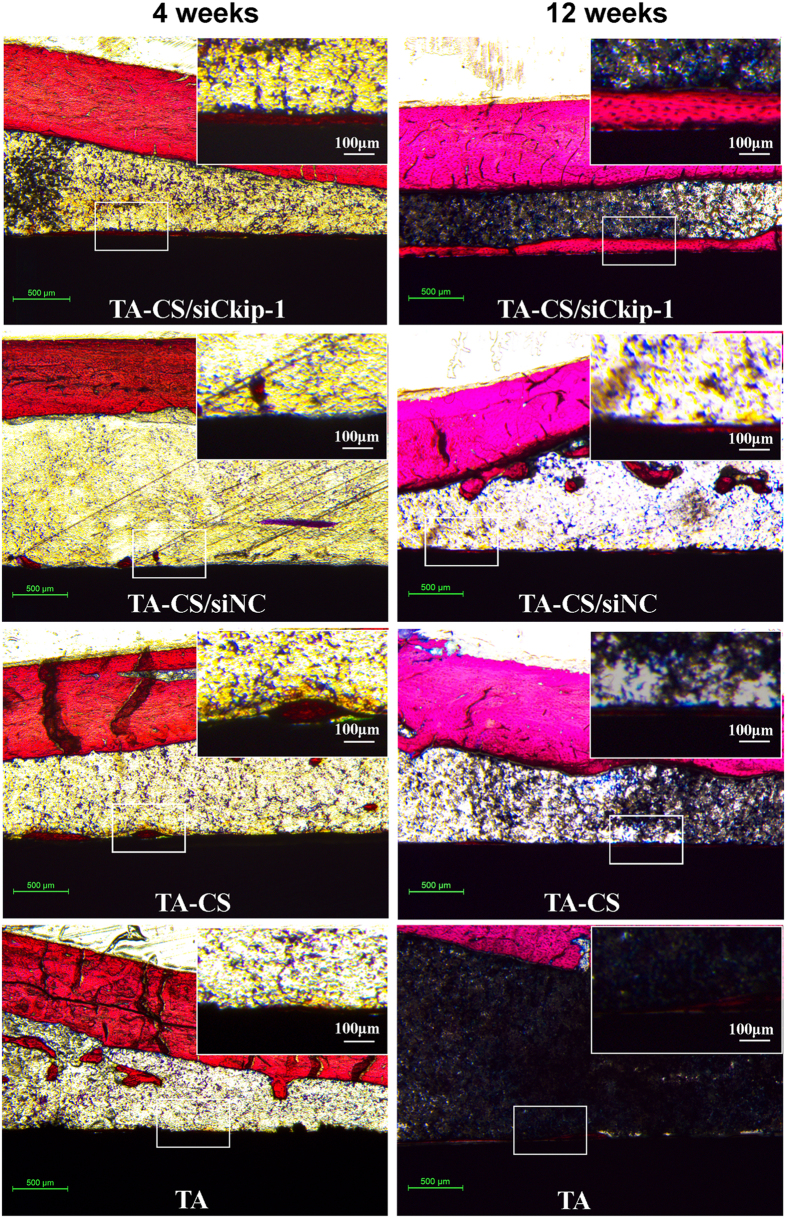
VG staining of the hard tissue sections after 4 and 12 weeks of implantation. . The upper right corner inset shows the higher magnification image of the part labeled by the white box.

The hard tissue sections with VG staining are exhibited in Fig 9. As early as 4 weeks after implantation, the bone formation on the controls was just sporadic, but that on TA-CS/siCkip-1 was continuous. With the further elongation of the healing time to 12 weeks, on the controls there was no obvious increase for the bone formation and meanwhile the bone continuity and bone-implant contact were not improved when compared to the results of 4 weeks. However, on TA-CS/siCkip-1 the volume of the newly formed bone increased significantly after 12 weeks to form a continuous and thick bone layer.

**Figure 10:**
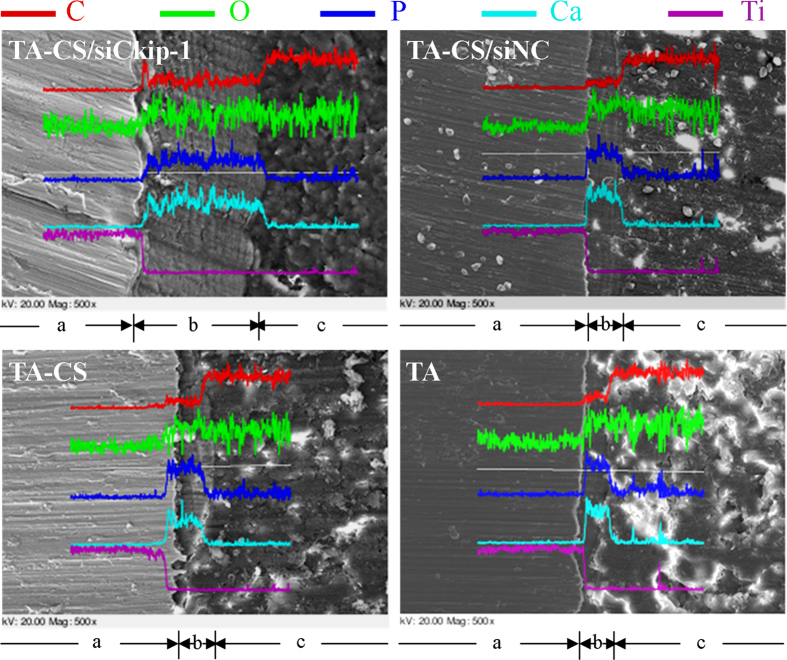
SEM inspection and EDX line scanning in the direction perpendicular to the bone/implant interface. (**a**: Ti implant; **b**: The Ca and P rich layer indexed as neo bone; **c**: Bone marrow cavity full-filled with methylmethacrylate).

The cross-section morphology of the bone/implant interface was inspected by SEM (Fig. 10). The distribution of the elements including Ti, C, O, Ca and P across the bone/implant interface was displayed by EDX line scanning. A good integration of the implants and the surrounding bone was observed without apparent disconnection. The line-scanning indicated that TA-CS/sickip-1 has the maximum range of newly formed bone around implants, as indexed by the Ca and P rich substance.

The maximal pull-out force and ultimate shear strength were used to index the bone/implant bonding strength (Table 1). As expected, the bone/implant bonding strength increased with the healing time from 4 to 12 weeks. TA-CS/siCkip-1 showed obviously higher maximal pull-out force and ultimate shear strength compared to the three controls at both time slots.

## Discussion

Advanced Ti implant with enhanced osseointegration in the osteoporotic condition is urgently in need and thus a hot topic of research^31^. Biofunctionalization with siRNA to target specific molecular events involved in the osteoporosis shall be an effective approach for developing such implant. In this study, TA-CS/siR was developed by the TA treatment followed by physical adsorption of the CS/siR complex, which gave rise to an even siRNA loading onto the microporous/nanofibrous Ti surface. TA-CS/siR showed excellent siRNA delivery efficiency and gene silencing effect. TA-CS/siCkip-1 targeting Ckip-1, a negative regulator of bone turnover, significantly improved the *in vitro* osteogenic differentiation of MSCs in terms of the enhanced ALP and collagen product and ECM mineralization, and led to dramatically enhanced *in vivo* osseointegration in the osteoporostic rat model, showing immense potential for achieving a satisfactory osseointegration in the osteoporotic condition.

Surface modification is the main strategy for obtaining implant of better performance, which is mainly divided into two categories, the inorganic modification on the surface morphological, chemical and hydrophilic properties and the organic modification that is biofunctionalization by immobilizing specific biomolecules^6^. The inorganically modified implants have proven their efficacy to achieve a good osseointegration in the normal bone condition and actually all the commercial implants belong to these kinds, however they are not bioactive enough to gain a rigid osseointegration in the osteoporotic condition that has a negative bone metabolism and a poor bone condition^4^. Relying on the effect of biomolecules, biofunctionalization can robustly change the bioinert Ti surface to be bioactive. Hitherto, the biomolecules used for biofunctionalization mainly include the extracellular matrix components, growth factors and peptides, which actually do not directly target the molecular mechanism of osteoporosis thus generating very limited effect^6^. The increasing understanding on the molecular events involved in osteoporosis and the quick advance in the siRNA delivery field provoke the idea of developing advanced implant specifically targeting the key molecular points in osteoporosis via siRNA biofunctionalization. There are several attempts on the siRNA biofunctionalization of polymer scaffolds 11,32 and we have tried the miRNA biofunctionalization Ti implant^15^,^16^, but the siRNA biofunctionalization of Ti implants has never been reported. In our previous report, the miRNA biofunctionalization of Ti was conducted on the Ti plates that were used for *in vitro* cell experiment via dropping the miRNA solution on to the Ti plate followed by lyophilisation^15^,^16^. This method is unable to form an even biofunctionalization on a 3-D target of complex shape, thus being unavailable for the 3-D cylindrical Ti implants. In this study, the TA treatment was applied to make the Ti surface ultrahydrophilic and negatively charged by increasing active OH groups^20^,^21^, which then facilitate an even loading of the positively charged CS/siR complex from the chitosan vector^22^ by simply immersing the Ti implant in the CS/siR complex solution. In addition, hydrogen bond may form between the active OH groups on Ti generated by the TA treatment and the amino groups rich in chitosan, leading to high binding strength. Meanwhile, the microporous/nanofibrous structure formed by the TA treatment significantly increases the loading capacity of Ti implant.

The successful clinical application of siRNA therapy requires both good safety and high delivery efficiency, which is mainly related to the delivery vector^33^ and delivery approach^25^. Due to the good biocompatibility, biodegradability, low toxicity and high ability to complexate the oligonucleotides, chitosan is believed to be a good candidate for gene delivery vector^33^. In spite of these excellent properties, the chitosan nanoparticle based delivery formulation generates low DNA/RNA delivery efficiency needing further augmentation^34^. According to previous report, the CS/siR nanoparticles are comprised of several chitosan molecules conjugated by siRNA serving as the bridge that due to the electrostatic force, which results in a wide size distribution and irregular morphology^35^. We have also observed the similar phenomenon in the DLS analysis. To improve the delivery efficiency, a lot of arduous work has been done on chemical modification of chitosan to obtain desirable physicochemical characteristics.^34^ Excitingly, efficient intracellular siRNA delivery and high gene knock down efficacy was observed from TA-CS/siR in this study. It is noted that the chitosan used in this study is the common one without any chemical modification. The high delivery efficiency can be attributed to the substrate-mediated reverse delivery, referring to the immobilization of delivery complex on a solid surface to deliver them locally to the attached cells^36^,^37^. Compared to the conventional delivery approach, the substrate-mediated reverse delivery enables a direct contact between the delivery agents and cells to improve the delivery efficiency^36^. In addition, it is indicated that suitable micro and nanotechnology on the substrate can improve endocytosis and thus oligonucleotide delivery efficiency^38^,^39^,^40^. Hence, the specific microporous/nanofibrous structure formed by the TA treatment may also contribute to the high delivery efficiency, while further study is necessitated to confirm this. A preliminary requirement for the clinical human body application of an implant is good cytocompatibility. The protein adsorption ability of an implant is important for its cytocompatibility^41^. We found that the TA treatment dramatically decreased the protein adsorption ability of Ti but excitingly TA-CS/siR showed enhanced protein adsorption ability even higher than the polished Ti control. The difference in protein adsorption might be related to the different charge of the Ti surface,^42^ while further study is required to verify this. The cell proliferation data indicated that TA-CS/siR could well support the growth of MSCs, demonstrating its good cytocompatibility. TA-CS/siR even showed higher cell number than the polished Ti control due to the TA treatment.

Choosing the proper gene knockdown target is pivotal for achieving satisfactory osteogenic activity for TA-CS/siR. After a thorough review of the reports on the mechanism of osteoporosis, we paid attention to Ckip-1, which is reported to act as a crucial suppressor of osteoblast differentiation and bone formation by positively regulating smurf1 and Rpt6^8^. The knockdown of Ckip-1 by siRNA induced bone mass increase in the osteoporotic rats,^9^,^10^,^43^ suggesting that Ckip-1 is a good therapeutic target for osteoporosis. Our *in vitro* data again confirmed the efficiency of siCkip-1 in promoting osteogenic differentiation and bone formation. TA-CS/siCkip-1 significantly improved the product of ALP that is the early marker of osteogenic differentiation, the secretion of collagen that is the main ECM component of bone, and ECM mineralization that is a key functional marker of mature osteoblasts during osteoblastogenesis.

Consistent with the *in vitro* results, enhanced peri-implant bone formation and osseointegration for TA-CS/siCkip-1 was observed in the *in vivo* osteoporotic model. The Micro-CT analysis, histological staining and line scanning of the bone-implant cross section jointly displayed that TA-CS/siCkip-1 gave rise to far better new bone formation than the controls. A continuous and thick layer of new bone was observed around TA-CS/siCkip-1, while that on the controls was sporadic. With the healing time from 4 to 12 weeks, there was no obvious increase for the bone formation on the controls, indicating their deficient bioactivity in the osteoporotic model. However, the new bone formation on TA-CS/siCkip-1 increased significantly with time to form a thick bone layer at 12 weeks, demonstrating its excellent osseointegration forming ability in the osteoporotic condition. Finally, the biomechanical strength of bone/implant integration was measured by the pull-out test. At both 4 and 12 weeks, TA-CS/siCkip-1 had the highest bone/implant strength. The good osseointegration of TA-CS/siCkip-1 in the osteoporotic rat model indicates its potential clinical application for the osteoporotic patients. Meanwhile, it is suggested that TA-CS/siCkip-1 might also be available for the patients of normal bone condition for enhanced clinical performance.

## Conclusions

Here the novel siRNA biofunctionalized Ti implant TA-CS/siR with an even siRNA loading was successfully developed by the TA treatment to make the Ti ultrahydrophilic and negatively charged to facilitate the physical adsorption of the positively charged CS/siR complex. TA-CS/siR showed excellent siRNA delivery efficiency and gene silencing effect. By targeting Ckip-1 that is a negative regulator of bone turnover, TA-CS/siCkip-1 significantly improved the *in vitro* osteogenic differentiation of MSCs and led to dramatically enhanced osseointegration in the *in vivo* osteoporostic rat model. TA-CS/siCkip-1 shows promising clinical potential for enhanced implant performance in the osteoporotic bone condition. Furthermore, TA-CS/siR shall provide a general approach for developing advanced Ti implants.

## Additional Information

**How to cite this article**: Zhang, L. *et al.* Chitosan/siCkip-1 biofunctionalized titanium implant for improved osseointegration in the osteoporotic condition. *Sci. Rep.* **5**, 10860; doi: 10.1038/srep10860 (2015).

## Supplementary Material

Supplementary Information

## Figures and Tables

**Table 1:** The maximal pull-out force and ultimate shear strength measured by pull-out test.

**Treatment**	**4 weeks**		**12 weeks**	
	**Maximal Pull-out force (N)**	**Ultimate shear Strength (N/mm**^2^)	**MaximalPull-out force (N)**	**Ultimate shear Strength (N/mm**^2^)
TA-CS/siCkip-1	46.67 ± 4.01*	1.09 ± 0.12^*^	93.05 ± 8.85^*^	2.17 ± 0.20^*^
TA-CS/siNC	33.90 ± 3.96	0.79 ± 0.07	56.30 ± 5.01	1.31 ± 0.11
TA-CS	32.07 ± 3.15	0.75 ± 0.07	54.03 ± 5.55	1.26 ± 0.12
TA	29.37 ± 2.63	0.68 ± 0.06	48.53 ± 4.17	1.13 ± 0.09

^*^p < 0.05
