# Supplementary material for: Chitosan/siCkip-1 biofunctionalized titanium implant for improved osseointegration in the osteoporotic condition
Source: Sci Rep. 2015 Jun 4;5:10860. doi: 10.1038/srep10860 (PMC4455222; doi:10.1038/srep10860)
Supplement: Supplementary Information [file srep10860-s1.doc]

**Chitosan/siCkip-1 biofunctionalized titanium implant for improved osseointegration in the osteoporotic condition**

*Li Zhang^1,5^, Kaimin Wu^3,5^, Wen Song^1^, Haiyan Xu^4^, Ran An^4^, Lingzhou Zhao^2*^, Bin Liu^4*^ and Yumei Zhang^1*^*

^1^State Key Laboratory of Military Stomatology, Department of Prosthetic Dentistry, School of Stomatology, The Fourth Military Medical University, No. 145 West Changle Road, Xi’an 710032, China

^2^State Key Laboratory of Military Stomatology, Department of Periodontology, School of Stomatology, The Fourth Military Medical University, No. 145 West Changle Road, Xi’an 710032, China

^3^Department of Stomatology, 401 Military Hospital, Qingdao 266071, China

^4^State Key Laboratory of Military Stomatology, Laboratory Animal Center, School of Stomatology, the Fourth Military Medical University, No. 145 West Changle Road, Xi’an 710032, China,

^5^Co-first authors

**Corresponding authors:*

*Prof. Yumei Zhang; E-mail: wqtzym@fmmu.edu.cn*

*Dr. Lingzhou Zhao; E-mail: zhaolingzhou1983@hotmail.com*

*Prof. Bin Liu; E-mail: kqyljd_liu@126.com*


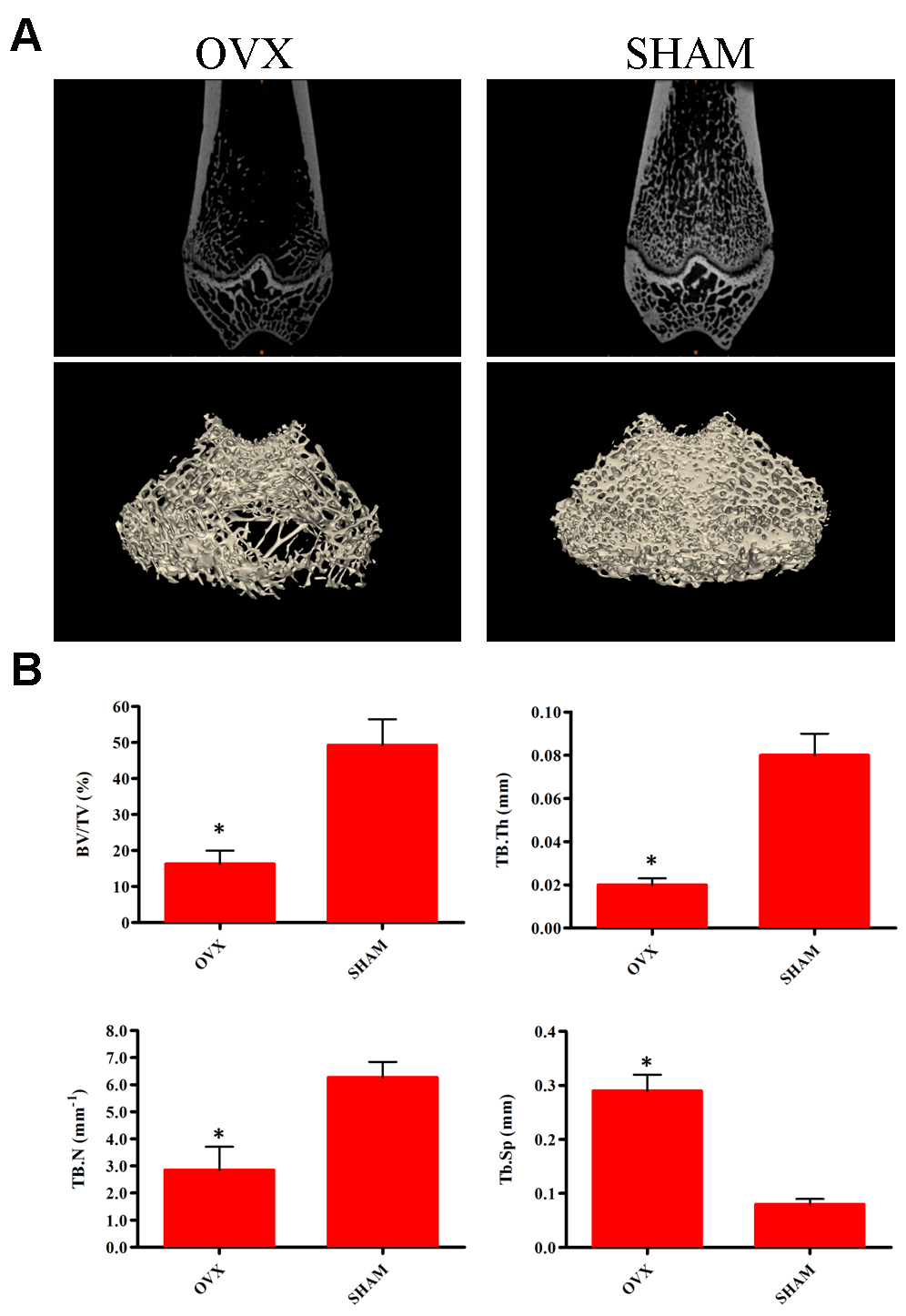


**Figure S1.** (A)Transverse 2-D images and 3-D reconstructed images of the distal femur metaphysis three months after bilateral ovariectomy. (B) The quantitative data obtained from the Micro-CT analysis including BV/TV, TB.Th, TB.N and TB.Sp. ^*^*p*<0.05 *vs* SHAM.
